# Reliability of serum neurofilament light and glial fibrillary acidic protein for detecting disease activity upon discontinuation of first-line disease-modifying therapy in stable multiple sclerosis (DOT-MS)

**DOI:** 10.1007/s00415-025-13231-9

**Published:** 2025-07-23

**Authors:** W. H. Fung, M. H. J. Wessels, E. M. E. Coerver, J. de Beukelaar, W. H. Bouvy, L. R. Canta, O. H. H. Gerlach, E. Hoitsma, E. L. J. Hoogervorst, B. A. de Jong, N. F. Kalkers, Z. L. E. van Kempen, H. Lövenich, C. E. P. van Munster, B. W. van Oosten, J. Smolders, A. Vennegoor, E. M. P. E. Zeinstra, M. Barrantes-Cepas, G. Kooij, M. M. Schoonheim, B. I. Lissenberg-Witte, B. Moraal, F. Barkhof, B. M. J. Uitdehaag, J. Mostert, C. E. Teunissen, J. Killestein, E. M. M. Strijbis

**Affiliations:** 1 MS Center Amsterdam, Neurology, Vrije Universiteit Amsterdam, Amsterdam Neuroscience, Amsterdam UMC location VUmc, De Boelelaan 1117, 1081 HV Amsterdam, The Netherlands; 2 Albert Schweitzer Hospital, Neurology, Dordrecht, The Netherlands; 3 Diakonessenhuis, Neurology, Utrecht, The Netherlands; 4 Catharina Hospital, Neurology, Eindhoven, The Netherlands; 5 Zuyderland Medical Center, Neurology, Sittard-Geleen, The Netherlands, and School for Mental Health and Neuroscience, Maastricht University, Maastricht, The Netherlands; 6 Alrijne Hospital, Neurology, Leiden, The Netherlands; 7 St. Antonius Hospital, Neurology, Utrecht, The Netherlands; 8 OLVG, Neurology, Amsterdam, The Netherlands; 9 St. Jans Gasthuis, Neurology, Weert, The Netherlands; 10 Amphia Hospital, Neurology, Breda, The Netherlands; 11 Erasmus Medical Center, MS center ErasMS, Neurology & Immunology, Rotterdam, The Netherlands; 12 Flevoziekenhuis, Neurology, Almere, The Netherlands; 13 Isala Hospital, Neurology, Meppel, The Netherlands; 14 MS Center Amsterdam, Anatomy and Neurosciences, Vrije Universiteit Amsterdam, Amsterdam Neuroscience, Amsterdam UMC, location VUmc, Amsterdam, The Netherlands; 15 MS Center Amsterdam, Molecular Cell Biology and Immunology, Vrije Universiteit Amsterdam, Amsterdam Neuroscience, Amsterdam UMC, location VUmc, Amsterdam, The Netherlands; 16 Department of Epidemiology and Data Science, Vrije Universiteit Amsterdam, Amsterdam UMC, Amsterdam, The Netherlands; 17 Radiology & Nuclear Medicine, MS Center Amsterdam, Vrije Universiteit Amsterdam, Amsterdam Neuroscience, Amsterdam UMC location VUmc, Amsterdam, The Netherlands; 18 Queen Square Institute of Neurology and Centre for Medical Image Computing, University College, London, UK; 19 Rijnstate Hospital, Neurology, Arnhem, The Netherlands; 20 Neurochemistry Laboratory, Department of Clinical Chemistry, Vrije Universiteit Amsterdam, Amsterdam Neuroscience, Amsterdam UMC, Amsterdam, The Netherlands

**Keywords:** MS, MRI in MS, Clinical trials

## Abstract

**Background:**

Neurofilament light(NfL) and glial fibrillary acidic protein(GFAP) are associated with disease activity in multiple sclerosis(MS), however use in monitoring remains limited. The ability of these biomarkers to detect disease activity upon treatment discontinuation was studied.

**Methods:**

Long-term stable relapse-onset MS patients were to continue or discontinue their first-line disease-modifying therapy(DMT) to study the safety of DMT discontinuation(DOT-MS trial NCT04260711). “Significant” disease activity was defined as clinical relapse, ≥3 new lesions or ≥2 contrast-enhancing lesions. MRI and sampling were performed at baseline, month 3, 6, 12, 18 and 24. Associations of delta biomarker levels and NfL z-score(age and body mass index derived) with “significant” disease activity were tested. Cut-off values for biomarkers to detect disease activity were calculated.

**Results:**

45(50.5%) participants discontinued their DMT. Eight(all discontinued DMT) had “significant” disease activity, which was associated with an increase in NfL levels(OR:1.13 [1.03–1.33], *p* = 0.04) and NfL *z*-scores(OR:2.17 [0.98–5.22], *p* = 0.06), but not with GFAP(*p* = 0.52). Delta NfL had the highest ability to detect “significant” disease activity(AUC:0.88 [0.76–0.99]), with the best calculated cut-off of 46.4% increase(AUC:0.68, sensitivity 0.57, specificity 0.96).

**Discussion:**

NfL may be useful to identify, but not predict, disease activity after DMT discontinuation in MS. GFAP levels were not discriminatory for disease activity.

**Supplementary Information:**

The online version contains supplementary material available at 10.1007/s00415-025-13231-9.

## Introduction

The key pathological features behind the multiple sclerosis (MS) disease pathogenesis are thought to be neuroinflammation, demyelination and neurodegeneration [1, 2]. Blood-based biomarkers appear to be able to provide a relatively cheap and easily available method of assessing, monitoring or even predicting such processes on a group level [3–5]. However, implementation of these biomarkers in clinical practice remains slow [6].

Two widely investigated blood-based biomarkers in the context of MS are neurofilament light (NfL) and glial fibrillary acidic protein (GFAP). NfL and GFAP are proteins found in neurons and astrocytes, respectively. NfL serves as a specific biomarker for neuronal damage, with increasing levels during relapses or new MRI lesions [7], and decreasing levels upon effective treatment [8]. Additionally, NfL levels hold prognostic value for disability accumulation, especially in relapsing-remitting MS [9, 10]. On the other hand, GFAP is considered as a biomarker of astrocytic damage and reactive astrogliosis, although its association with acute disease activity is inconsistent in blood and cerebrospinal fluid samples [11, 12].

To use NfL and GFAP as tools for individual disease monitoring, standardized cut-off values still need to be established. Although fixed biomarker cut-offs for pathology and reference values correcting for age and Body Mass Index (BMI) have been explored previously, the individual use of NfL and GFAP has not been widely implemented as a clinically usable biomarker [13–16]. In our DOT-MS cohort (NCT04260711), we therefore investigated the ability of NfL and GFAP to discriminate disease activity and stable disease in participants with long-term stable MS who continued or discontinued first-line disease-modifying therapy (DMT).

## Methods

### Study design and participants

Eighty-nine participants were included from the DOT-MS trial [17]: an investigator-initiated, multicenter clinical trial, with randomized treatment-group assignments, open-label treatment and blinded end-point evaluation. Participants were randomized into continuation of DMT or discontinuation of DMT. Participants were 18 years or older, had relapse-onset MS, used first-line DMT (any of the interferons, glatiramer acetate, teriflunomide or dimethyl fumarate), and no clinical relapses nor substantial radiological disease activity (defined as no new contrast-enhancing lesions and ≤1 new T2 lesion on MRI) for at least 5 years before inclusion.

### Study procedures and outcomes

Participants underwent a clinical evaluation, brain MRI with gadolinium and blood sampling at baseline, 3, 6, 12, 18 and 24 months. At each visit, all assessments were performed on the same day. In addition to the scheduled visits, an unscheduled visit was arranged if deemed necessary by the physician. MRI assessments were performed by neuro-radiologists blinded to treatment allocation. In addition, scans that showed any MRI activity were centrally reviewed by a dedicated neuroradiologist in the main investigation center (Amsterdam UMC, location VUmc). Spinal cord imaging was only performed in relapses with suspected spinal cord involvement.

“Significant” disease activity was defined as any confirmed clinical relapse and/or “significant” MRI activity. “Significant” MRI activity was defined as ≥3 new T2 lesions or ≥2 contrast-enhancing lesions on brain MRI. In addition, we included “any” MRI activity, defined as “significant” disease activity and any other MRI activity (such as one or two new T2 lesions, one contrast-enhancing lesion or enlarged T2 lesion).

### Biomarker analysis

Blood samples were collected at baseline and all follow-up study visits. Blood was centrifuged at 1800 g for 10 minutes at room temperature, aliquoted, and stored at −80°C. Serum NfL and GFAP levels were assessed using the Simoa™ N4PE kit on the Simoa™ HD-X instrument following the instructions (Quanterix, Billerica, USA) as described in detail previously [18–20]. Intra- and inter-assay precision of three quality control samples measured in duplicate over four runs ranged for NfL respectively from 7.0% to 8.7% and 7.0% to 9.8%, and for GFAP 4.2% to 12.2% and 7.7% to 12.2%.

We calculated absolute changes in NfL (absolute delta NfL) and GFAP (absolute delta GFAP) levels as the absolute difference between the NfL and GFAP levels in comparison to the previous measurement. Similarly, percentage changes in NfL and GFAP levels were calculated by determining the percentage difference between the NfL (percentage delta NfL) and GFAP (percentage delta GFAP) levels compared to those from the previous measurement. Lastly, for age and BMI-adjusted normative NfL values, z-scores were derived from the online application created by Benkert et al. [15] Absolute delta NfL levels were adjusted for BMI, except in the analysis involving cut-off values, where the absolute delta NfL levels were not BMI-adjusted.

### Statistical analysis

**Fig. 1:**
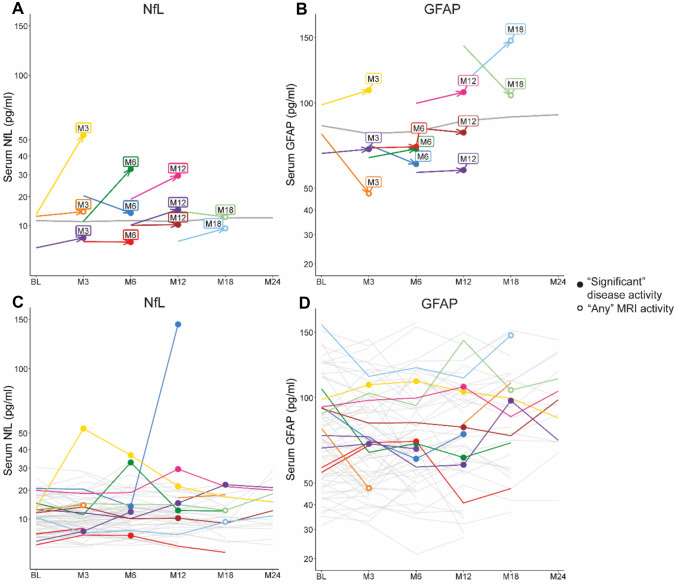
**A**, **B** The mean change in NfL and GFAP at time of first occurrence of “significant” disease activity or “any” MRI activity. The grey line represents the overall mean change in NfL and GFAP levels in participants without such disease activity. **C**, **D** Individual longitudinal trajectories of NfL and GFAP levels in MS. Absolute serum NfL and GFAP levels are presented

Statistical analysis was performed using R version 4.2.1. A *p* value ≤ 0.05 was considered statistically significant. Two-sample *t*-test and the Mann–Whitney test were used appropriately based on visual inspection of data distribution. Proportions were compared using the Chi-square test. To study the associations between delta NfL levels, delta GFAP levels and NfL z-score with “significant” disease activity and “any” MRI activity, we performed logistic regression analysis. The mean delta NfL levels, delta GFAP levels and NfL *z*-score at the time of first occurrence of “significant” disease activity or “any” MRI activity in those participants were compared with mean delta NfL levels, delta GFAP levels and NfL z-score over time in participants without such activity (Fig. 1A, B). Comparison of delta biomarker changes at first activity versus at last sampling in patients without activity are available as supplementary material. We calculated the odds of an event (“significant” disease activity or “any” MRI activity) at follow-up for delta NfL levels, delta GFAP levels and NfL *z*-score. Covariates (BMI, age, sex) were separately and only jointly entered into the delta NfL and delta GFAP model, and the best fitting model was then selected based on the likelihood ratio tests. sNfL values were predicted with each model and compared to actual sNfL values using the Hosmer-Lemeshow test, after which a receiver operating characteristic (ROC) curve was used to assess sensitivity and specificity of each model for “significant” disease activity and “any” MRI activity. Cut-off values were selected from univariate models using Youden’s *J* statistic. The proposed cut-off value of the NfL *z*-score of >1.5 for a significantly higher chance of clinical and/or radiological activity [15] was also considered. To evaluate the magnitude of NfL elevations, we calculated the 95th percentile threshold for percentage change from baseline in the participants who did not develop “significant” disease activity and “any” MRI activity. This threshold was then used to evaluate the magnitude of NfL elevations in participants who did develop disease activity.

In addition, we have investigated the association between GFAP changes and confirmed disability progression. Significant confirmed disability progression was defined as an increase of ≥1.0 point from the baseline EDSS score if the baseline score was ≤5.5 or an increase of ≥0.5 points if the baseline score was >5.5, sustained for at least 24 weeks. The change of GFAP levels for participants with confirmed disability progression was analyzed using 1-sample *t* test.

### Standard protocol approvals, registrations, and patient consents

Written informed consent was obtained from all participants. The use of data for this study was approved by the Medical Ethical Committee of the Amsterdam UMC, location VUmc (Protocol ID NL71260.029.19; clinicaltrials.gov identifier NCT04260711).

### Data availability

Anonymized data not published within this article will be made available by request from any qualified investigator.

## Results

Out of 89 participants (*n* = 44 in the continue group and *n* = 45 in the discontinue group), eight participants had “significant” disease activity, all in the discontinue group, as previously described [17]. There were four additional participants with “any” MRI activity other than “significant” disease activity. Thus, there were in total 12 participants with “any” MRI activity, one of which was in the continue group and 11 in the discontinue group. No participants experienced clinical relapse without inflammatory MRI activity.

The participants had a median follow-up of 15.3 months (IQR 11.4–23.9). Not all participants completed the 24-month follow-up due to premature termination of the trial: all cases of “significant” disease activity were observed in the discontinuation group, with no cases as such observed in the continuation group.

### Serum NfL and GFAP levels

Baseline NfL and GFAP levels did not differ between the continue and discontinue group (median NfL 10.53 [IQR 7.78–14.91] vs 10.27 [IQR 6.37–14.47], *p* = 0.59; median GFAP 74.28 [IQR 53.67–109.62] vs 77.78 [IQR 58.55-94.94], *p* = 0.90; Table 1). Also, no differences in baseline NfL or GFAP levels were found between participants with and without “significant” disease activity or “any” MRI activity (Table 1). Mean NfL levels of participants at visits during “significant” disease activity were higher than mean NfL levels at all visits of participants without “significant” disease activity (median NfL 18.31 [IQR 12.49–30.33] vs 10.37 [IQR 7.58–14.29], *p* < 0.001). Additionally, mean NfL levels of participants at visits during “any” MRI activity were higher compared to mean NfL levels at all visits of participants without “any” MRI activity (median NfL level 13.94 [IQR 12.14–29.50] vs 10.27 [IQR 7.56–14.01], *p* = 0.002). For the NfL change compared to the previous measurement, participants with “significant” disease activity or “any” MRI activity had a higher increase in their NfL level at the first occurrence of disease activity relative to the prior measurement compared to the participants without disease activity (Table 2). Absolute GFAP levels nor changes in GFAP were different between participants with or without “significant” disease activity or “any” MRI activity. Fig. 1C, D shows the longitudinal trajectories of NfL and GFAP levels per participant.

**Table 1.** Baseline characteristics of the study cohort

	All participants (*n* = 89)	“Significant” disease activity (*n* = 8)	No “significant” disease activity (*n* = 81)	“Any” MRI activity (*n* = 12)	No “any” MRI activity (*n* = 77)
Median age (IQR), years	54 (49.0-59.0)	46 (43.5-58.5)	54 (50.0-59.0)	50 (43.5-60.25)	54 (50.0-58.0)
Sex					
Female	60 (67.4%)	5 (62.5%)	55 (68.0%)	9 (75.0%)	51 (66.2%)
Male	29 (32.6%)	3 (37.5%)	26 (32.0%)	3 (25.0%)	26 (33.8%)
Median time since symptom onset (IQR), years	14.0 (9.9-21.5)	14.5 (13.2-16.4)	14.0 (9.5-21.6)	13.5 (11.2-15.5)	14.2 (9.9-23.3)
Median time since last documented relapse (IQR), years	9.4 (6.9-13.2)	9.6 (7.4-11.2)	9.5 (6.9-13.2)	10.4 (8.1-12.3)	9.2 (8.1-12.3)
Multiple sclerosis subtype					
Relapsing-remitting	80 (89.9%)	8 (100.0%)	72 (88.9%)	12 (100.0%)	68 (88.3%)
Secondary progressive	9 (10.1%)	0 (0.0%)	9 (11.1)	0 (0.0%)	9 (11.7%)
Median total duration of disease-modifying therapy use (IQR), years	11.2 (7.7-16.0)	10.3 (8.8-11.4)	11.4 (7.7-16.2)	10.4 (8.1-12.3)	9.4 (6.8-13.2)
Disease-modifying therapy at randomisation					
Interferon beta	35 (39.3%)	2 (25.0%)	33 (40.7%)	3 (25.0%)	32 (41.5%)
Glatiramer acetate	23 (25.8%)	1 (12.5%)	22 (27.2%)	3 (25.0%)	20 (26.0%)
Teriflunomide	12 (13.5%)	2 (25.0%)	10 (12.3%)	2 (16.7%)	10 (13.0%)
Dimethyl fumarate	19 (21.3%)	3 (27.5%)	16 (19.8%)	4 (33.3%)	15 (19.5%)
Expanded Disability Status Scale score	3.1 (1.8)	3.3 (0.9)	3.1 (1.8)	2.9 (1.0)	3.1 (1.9)
Median neurofilament light chain (IQR), pg/mL	10.3 (7.1-14.9)	13.1 (10.0-16.3)	10.1 (7.1-14.4)	12.5 (9.3-13.8)	10.0 (7.1-14.9)
Median glial fibrillary acidic protein (IQR), pg/mL	75.0 (55.8-97.7)	93.2 (73.8-94.9)	73.6 (54.4-109.6)	91.1 (73.8-94.9)	72.4 (53.7-109.6)
Median follow-up (IQR), months	15.3 (11.4-23.9)	21.5 (18.0-24.1)	13.8 (10.3-23.7)	21.5 (18.0-24.1)	13.6 (9.2-23.7)
Median follow-up (IQR) till disease activity, months		12.0 (6.75-12.0)		12.0 (6.0 - 12.0)	

Data are *n* (%) or mean (SD), unless otherwise specified. “Significant” disease activity was defined as any confirmed relapse

and/or “significant” MRI activity (≥3 new T2 lesions or ≥2 contrast-enhancing lesions on brain MRI). “Any” MRI activity was defined as any new T2 lesions, contrast-enhancing lesions or enlarged T2 lesions in addition “significant” disease activity

**Table 2.** Change in absolute serum NfL and GFAP levels at follow-up

	All participants (*n* = 89)	No “significant” disease activity (*n* = 81)	No “any” MRI activity (*n* = 77)
Median delta NfL (IQR), pg/mL	0.05 (−0.61–0.62)	0.02 (−0.63–0.52)	0.01 (−0.67–0.52)
Median delta GFAP (IQR), pg/mL	−0.43 (−3.61–3.80)	−0.43 (−3.61–3.80)	−0.43 (−3.87–3.58)
Median NfL z-score (IQR)	0.32 (−0.35–0.99)	0.32 (−0.36–0.96)	0.32 (−0.39–0.94)

	“Significant” disease activity (*n* = 8)	“Any” MRI activity (*n* = 12)
Median delta NfL during disease activity (IQR), pg/mL	2.14 (0.03–13.3)	2.14 (0.03–7.72)
Median delta GFAP during disease activity (IQR), pg/mL	1.20 (−1.07–3.74)	1.20 (−6.82–6.41)
Median NfL z-score during disease activity	0.64 (0.09–1.87)	0.64 (0.14–1.64)

Delta NfL and delta GFAP were calculated as the absolute change in NfL and GFAP levels compared to the previous measurement during the whole follow-up. For “significant” disease activity and “any” MRI activity, the delta NfL and delta GFAP were calculated as the absolute change in NfL and GFAP levels compared to the previous measurement at the time of first occurrence of disease activity

*NfL* neurofilament light chain, *GFAP* glial fibrillary acidic protein

Absolute delta NfL levels, corrected for BMI (OR: 1.13, 95% CI [1.03–1.33], *p* = 0.04), and percentage delta NfL levels (OR: 1.02, 95% CI [1.01–1.03], *p* = 0.01) were associated with “significant” disease activity. For “any” MRI activity, there was a significant association observed with percentage delta NfL levels (OR: 1.02, 95% CI [1.01–1.03], *p* = 0.01) and NfL z-score (OR: 2.08, 95% CI [1.07–4.30], *p* = 0.04) but not with absolute delta NfL levels. In contrast, neither absolute delta GFAP levels, or percentage delta GFAP levels were significantly associated with “significant” disease activity or “any” MRI activity (Table 3).

**Table 3.** Logistic regression and sensitivity and specificity of NfL and GFAP

	Odds ratio [95% CI]	*p*-value*	AUC [95% CI]	Cut-off**	Sensitivity	Specificity
NfL in “significant” disease activity						
Absolute delta	1.13 [1.03–1.33]	0.04	0.88 [0.76–0.99]	≥2.14	0.57	0.89
Percentage delta	1.02 [1.01–1.03]	0.01	0.68 [0.38–0.98]	≥46.4	0.57	0.96
*Z*-score	2.17 [0.98–5.22]	0.06	0.63 [0.38–0.87]	≥2.79	0.29	1.00
NfL in “any” MRI activity						
Absolute delta	1.13 [1.02–1.33]	0.06	0.83 [0.70–0.97]	≥1.55	0.64	0.87
Percentage delta	1.02 [1.01–1.03]	0.01	0.70 [0.47–0.93]	≥46.4	0.55	0.96
*Z*-score	2.08 [1.07–4.30]	0.04	0.63 [0.44–0.81]	≥2.33	0.27	1.00
GFAP in “significant” disease activity						
Absolute delta	1.04 [0.95–1.20]	0.52	0.57 [0.34–0.80]			
Percentage delta	1.00 [0.94–1.08]	0.99	0.51 [0.29–0.74]			
GFAP in “any” MRI activity						
Absolute delta	1.00 [0.95–1.06]	0.90	0.55 [0.32–0.78]			
Percentage delta	0.98 [0.94–1.04]	0.53	0.50 [0.28–0.73]			

“Significant” disease activity was defined as any confirmed relapse and/or “significant” MRI activity (≥3 new T2 lesions or ≥2 contrast-enhancing lesions on brain MRI). “Any” MRI activity was defined as any new T2 lesions, contrast-enhancing lesions or enlarged T2 lesions in addition “significant” disease activity

^*^p-value from logistic regression

^**^Cut-off values were calculated univariate using Youden’s J statistic and not corrected for BMI

*NfL* neurofilament light chain, *GFAP* glial fibrillary acidic protein, *AUC* area under the Curve

### Sensitivity and specificity analyses

**Fig. 2:**
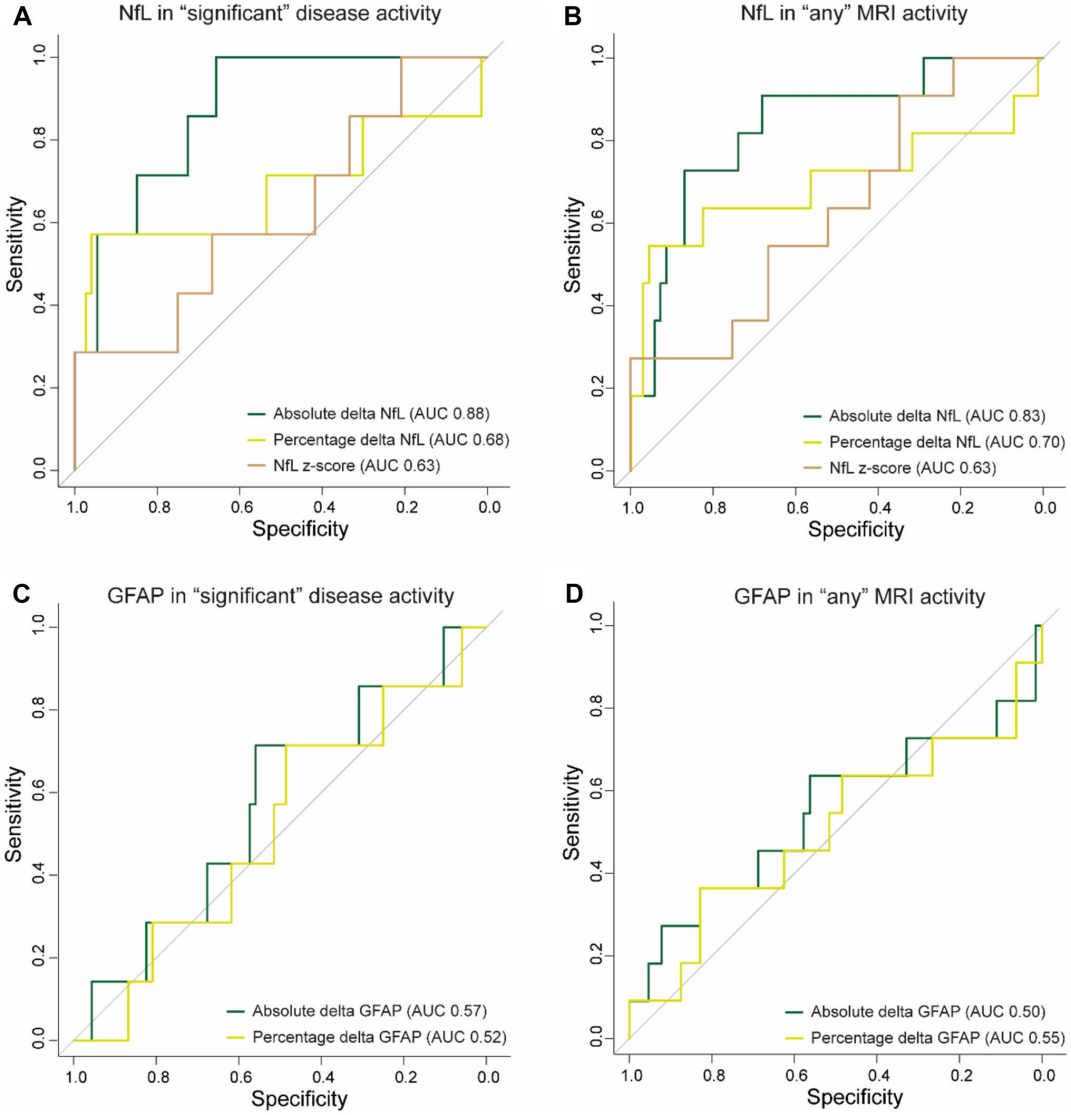
Receiver operating characteristic curves for NfL and GFAP in those with “significant’ disease activity and “any” MRI activity. Absolute delta NfL models were corrected for BMI

ROC curves were calculated for absolute delta NfL and GFAP levels, percentage delta NfL and GFAP levels, and NfL z-score for both “significant” disease activity” and “any” MRI activity. The model with absolute delta NfL change corrected for BMI performed best compared to percentage delta NfL or NfL z-score at the first moment of inflammatory disease activity, both in detecting “significant” disease activity (AUC 0.88, 95% CI [0.76–0.99]) and “any” MRI activity (AUC 0.83, 95% CI [0.70–0.97]) (Figure 2). In contrast, absolute delta GFAP levels and percentage delta GFAP level change were not able to detect “significant” disease activity or “any” MRI activity.

### Serum NfL cut-off value for inflammatory disease event

Univariate and uncorrected cut-off values for NfL levels in this cohort were calculated using Youden’s J statistic (Table 2). For “significant” disease activity, a cut-off was calculated in percentage increase in NfL level (≥46.4%, AUC 0.68, 95% CI [0.38–0.98], sensitivity 0.57, specificity 0.96, positive predictive value 0.57, negative predictive value 0.96). For “any” MRI activity, a cut-off was calculated in percentage increase in NfL level (≥46.4%, AUC 0.70, 95% CI [0.47–0.93], sensitivity 0.55, specificity 0.96, positive predictive value 0.67, negative predictive value 0.93) and NfL *z*-score at moment of activity (≥2.33, AUC 0.63, 95% CI [0.38–0.87], sensitivity 0.27, specificity 1.00, positive predictive value 1, negative predictive value 0.89). A previously proposed cut-off of *Z*-score ≥1.5 [15] performed comparably (AUC 0.63, sensitivity 0.27, specificity 0.90, positive predictive value 0.3, negative predictive value 0.89).

### Changes in NfL levels prior first occurrence of disease activity

The 95th percentile threshold of percentage change from baseline was 46.47% for “significant” MRI activity. For “any” MRI activity, the 95th percentile threshold of percentage change from baseline was 45.85%. Only 1/8 participants with “significant” disease activity and 1/12 participants with “any” MRI activity t exceeded the 95th percentile threshold for percentage change from baseline.

### GFAP and confirmed disability progression

There were no associations between GFAP levels and clinical disability. The change of GFAP levels between baseline and confirmed disability progression was 0.72% (SD 9.28; *p* = 0.83), and the change between baseline and the visit prior confirmed disability progression was −2.45% (SD 11.98; *p* = 0.58).

## Discussion

Serum neurofilament light is known to be associated with disease activity, and has been used and proposed as a treatment response biomarker [7, 8]. Investigation into the clinical application of biomarkers in context of de-escalation is ongoing [21, 22]. In this study, we aimed to evaluate the utility of serum NfL and GFAP levels to discriminate participants with disease activity and stable disease. In the DOT-MS cohort, participants with “significant” disease activity and “any” MRI activity showed a clear increase in their NfL levels, while this was not apparent for GFAP levels. We did not see a difference in biomarker levels before disease activity. Based on the models’ performance, NfL levels were useful to detect disease activity due to high specificity, while GFAP levels were not discriminative of disease activity or stable disease.

Absolute change of serum NfL, corrected for BMI, best detected “significant” disease activity and “any” MRI activity at that timepoint, with the highest performance reached for “significant” disease activity. This could possibly be explained by higher NfL levels in more active disease states as it has been shown that higher serum NfL levels correlate with the number and volume of contrast-enhancing lesions, and T2 lesion volume [23]. It should be noted that in all models involving NfL levels, the sensitivity for detecting a disease event is relatively low compared to a higher specificity. NfL could detect disease activity after DMT discontinuation and identify disease activity when a clinical relapse is suspected. This could help early recognition of the recurrence of significant inflammatory disease activity. However, NfL is less useful to rule out disease activity, and, therefore, clinical and radiological monitoring after DMT discontinuation is still necessary. In line with a previous study, NfL also had somewhat lower sensitivity and higher specificity to detect contrast-enhancing lesions in people with MS who continued or discontinued natalizumab. Next to that, the model for disease activity, including sNfL z-scores previously calculated underperformed in this cohort [15]. This could imply that standardized NfL z-scores may not universally apply to all individuals, while individual changes in NfL may be more indicative of disease activity. Moreover, the consideration of assay variability should be factored into the utilization of NfL z-score as a tool to detect disease activity. In addition, we have now defined criteria for “significant” disease activity and “any” MRI activity, but future research should also consider further categorization of disease activity into new T2 lesions, contrast-enhancing lesions, enlarging T2 lesions and clinical relapses, while also investigating the role of NfL in this context.

In the DOT-MS trial, we highlight that serum biomarkers (NfL and GFAP) did not predict subsequent later disease activity, consistent with findings from a prior study examining the temporal association between NfL levels and new disease activity [24]. Similarly, that study reported low sensitivity but high specificity of NfL in detecting disease activity, aligning with our results. In contrast, another study has shown that either NfL or GFAP following treatment discontinuation were associated with an increased risk of 6-month confirmed disability worsening and the development of new MRI activity [22]. While we also found a significant association between increased NfL levels and new disease activity, this relationship was not observed for GFAP. A possible explanation for this discrepancy is the difference in study design: the referenced study collected biomarker samples within two years following treatment discontinuation and assessed outcomes at follow-up several years later. In contrast, our analysis focused on the association between biomarker levels and new disease activity over a shorter period, within a 3–6-month time window. Additionally, the referenced study identified associations between biomarker levels and both disease activity (median 5.15 years after discontinuation) and confirmed disability progression (median 1.99 years after discontinuation). Unfortunately, our follow-up period is not yet long enough to capture such long-term outcomes. However, as the DOT-MS trial continues in an observational phase for an additional two years, future analyses will also focus on the association between biomarker levels and disease recurrence and confirmed disability progression at a longer follow-up duration.

Our study has limitations. Due to the early termination of the trial, the sample size of the DOT-MS trial was smaller than calculated in the preliminary power calculation: 45 and 45 versus the necessary sample size of 54 per group to achieve 80% power [17]. This resulted in a limited number of events. However, despite this limitation, we were still able to demonstrate the ability of NfL in identifying disease activity. Additionally, since blood sampling and MRI scans were conducted on the same day of the visit, assessing the predictive value of NfL for disease activity is challenging. To address this, we calculated the 95th percentile threshold for the percentage change from baseline in participants who did not develop disease activity, allowing us to evaluate the magnitude of NfL level elevations [23]. Factors such as renal function and other comorbidities, known to influence NfL levels, were not accounted for in the sensitivity and specificity analyses [6]. In addition, NfL is also a non-specific MS marker, and may indicate other neuronal pathology. Our analyses were not corrected for the randomization group (continuation vs discontinuation of DMT) of the participants as the longitudinal NfL levels did not differ between discontinuation and continuation of DMT [17]. We suggest future research exploring NfL and its cut-offs further in a bigger cohort to validate our current findings. The DOT-MS cohort had a relatively older median age and remained stable over a long period. Future studies focusing on the NfL and GFAP course in the younger population are still needed.

In summary, absolute change of NfL levels corrected for BMI could serve as valuable tool for identifying and monitoring, but not predicting, disease activity in treatment discontinuation in MS. For now it remains a tool with caveats: the time between activity and NfL peaks is uncertain, NfL misses clear cut-off values for one-off measurements and clinical experience is still in its infancy [21, 24, 25]. While NfL levels alone may not be sufficient, the combination of NfL with clinical and MRI could enhance the ability and efficiency of identifying disease activity. Repeated NfL measurements could thus be used in adjunct to the necessary close clinical and radiological monitoring after discontinuation of a patient’s DMT. This data contributes to the evaluation of NfL’s role in regular MS care.

## Supplementary Information

Below is the link to the electronic supplementary material.

Supplementary file1 (DOCX 29 KB)

Supplementary file2 (DOCX 31 KB)
