# Supplementary material for: Reliability of serum neurofilament light and glial fibrillary acidic protein for detecting disease activity upon discontinuation of first-line disease-modifying therapy in stable multiple sclerosis (DOT-MS)
Source: J Neurol. 2025 Jul 23;272(8):530. doi: 10.1007/s00415-025-13231-9 (PMC12287240; doi:10.1007/s00415-025-13231-9)
Supplement: Supplementary file 1 — Supplementary file1 (DOCX 29 KB) [file 415_2025_13231_MOESM1_ESM.docx]

| **Supplementary table 1. Change in absolute serum NfL and GFAP levels at first activity versus at last sampling.** | | |
| --- | --- | --- |
|  | No “significant” disease activity (n=81) | No “any” MRI activity (n=77) |
| Median delta NfL (IQR), pg/mL | *0.00 (-1.59-1.41)* | *-0.15 (-1.70-1.41)* |
| Median delta GFAP (IQR), pg/mL | 0.51 (-3.54-4.84) | -0.33 (-3.80-3.76) |
| Median NfL z-score (IQR) | 0.33 (-0.34-1.13) | 0.33 (-0.36-1.64) |
|  | “Significant” disease activity (n=8) | “Any” MRI activity (n=12) |
| Median delta NfL during disease activity (IQR), pg/mL | 2.14 (0.03-13.3) | 2.14 (0.03-7.72) |
| Median delta GFAP during disease activity (IQR), pg/mL | 1.20 (-1.07-3.74) | 1.20 (-6.82-6.41) |
| Median NfL z-score during disease activity | 0.64 (0.09-1.87) | 0.64 (0.14-1.64) |
| For "significant" disease activity and "any" MRI activity, the delta NfL and delta GFAP were calculated as the absolute change in NfL and GFAP levels compared to the previous measurement at the time of first occurrence of disease activity. For no activity, delta NfL and delta GFAP were calculated as the absolute change in NfL and GFAP levels compared to the previous measurement at the time of last sampling. NfL = Neurofilament light chain, GFAP = Glial Fibrillary Acidic Protein. | | |
