# Supplementary material for: Reliability of serum neurofilament light and glial fibrillary acidic protein for detecting disease activity upon discontinuation of first-line disease-modifying therapy in stable multiple sclerosis (DOT-MS)
Source: J Neurol. 2025 Jul 23;272(8):530. doi: 10.1007/s00415-025-13231-9 (PMC12287240; doi:10.1007/s00415-025-13231-9)
Supplement: Supplementary file 2 — Supplementary file2 (DOCX 31 KB) [file 415_2025_13231_MOESM2_ESM.docx]

| **Supplementary table 2. Logistic regression and sensitivity and specificity of NfL and GFAP** | | | | | | |
| --- | --- | --- | --- | --- | --- | --- |
| **NfL in "significant" disease activity** | | | | | | |
|  | Odds ratio [95% CI] | P-value* | AUC [95% CI] | Cut-off** | Sensitivity | Specificity |
| Absolute delta | 1.07 [1.00-1.17] | 0.04 | 0.85 [0.72-0.98] | ≥2.14 | 0.57 | 0.81 |
| Percentage delta | 1.01 [1.00-1.01] | 0.08 | 0.69 [0.43-0.95] | ≥46.4 | 0.57 | 0.92 |
| Z-score | 1.63 [0.84-3.25] | 0.15 | 0.63 [0.39-0.87] | ≥0.64 | 0.57 | 0.69 |
| **NfL in "any" MRI activity** |  |  |  |  |  |  |
| Absolute delta | 1.07 [0.99-1.14] | 0.08 | 0.81 [0.67-0.95] | ≥1.55 | 0.64 | 0.77 |
| Percentage delta | 1.00 [1.00-1.01] | 0.11 | 0.70 [0.51-0.89] | ≥46.4 | 0.55 | 0.91 |
| Z-score | 1.57 [0.91-2.79] | 0.11 | 0.63 [0.46-0.81] | ≥-0.05 | 0.91 | 0.36 |
| **GFAP in "significant" disease activity** | |  |  |  |  |  |
| Absolute delta | 1.00 [0.96-1.00] | 0.64 | 0.54 [0.33-0.75] |  |  |  |
| Percentage delta | 1.00 [0.95-1.00] | 0.72 | 0.51 [0.30-0.73] |  |  |  |
| **GFAP in "any" MRI activity** |  |  |  |  |  |  |
| Absolute delta | 1.00 [0.96-1.06] | 0.94 | 0.54 [0.32-0.77] |  |  |  |
| Percentage delta | 0.99 [0.95-1.04] | 0.62 | 0.50 [0.28-0.72] |  |  |  |
| Delta NfL and delta GFAP was defined as the absolute change in NfL and GFAP levels compared to the previous measurement at the time of first activity versus at last sampling in patient without activity. “Significant” disease activity was defined as any confirmed relapse and/or “significant” MRI activity (≥3 new T2 lesions or ≥2 contrast-enhancing lesions on brain MRI). "Any" MRI activity was defined as any new T2 lesions, contrast-enhancing lesions or enlarged T2 lesions in addition "significant" disease activity. *P-value from logistic regression. **Cut-off values were calculated univariate using Youden’s J statistic and not corrected for body mass index. NfL = Neurofilament light chain, GFAP = Glial Fibrillary Acidic Protein, AUC = Area under the Curve. | | | | | | |
